# Biomechanical Analysis of Diffuse Idiopathic Skeletal Hyperostosis and Osteoporosis: Vertebral Fracture Risk Evaluation Using Finite Element Modeling with Clinical and Micro-CT Data in an Elderly Donor

**DOI:** 10.3390/biomedicines12112496

**Published:** 2024-10-31

**Authors:** Guido Schröder, Thomas Mittlmeier, Steffi S. I. Falk, Andreas Götz, Josephine Kruse, Estelle Akl, Hannes Kröger, Julian Ramin Andresen, Reimer Andresen, Hans-Christof Schober, Änne Glass

**Affiliations:** 1 Department of Traumatology, Hand and Reconstructive Surgery, Rostock University Medical Center, Schillingallee 35, 18057 Rostock, Germany; thomas.mittlmeier@med.uni-rostock.de (T.M.); steffi.falk@med.uni-rostock.de (S.S.I.F.); 2 Institute for Biomedical Engineering, Rostock University Medical Center, Friedrich-Barnewitz-Strasse 4, 18119 Rostock, Germany; andreas.goetz@med.uni-rostock.de; 3 Faculty of Medicine, University of Rostock, Ernst-Heydemann-Str. 8, 18057 Rostock, Germany; josephine.kruse@uni-rostock.de; 4 Institute for Diagnostic and Interventional Radiology, Pediatric and Neuroradiology, Rostock University Medical Center, Schillingallee 35, 18057 Rostock, Germany; estelle.akl@med.uni-rostock.de; 5 Silent Dynamics, Joachim-Jungius-Straße 9, 18059 Rostock, Germany; hannes.kroeger@silentdynamics.de; 6 Division of Trauma Surgery, Department of Orthopaedics and Trauma Surgery, Medical University of Vienna, Währinger Gürtel 18-20, 1090 Vienna, Austria; julian.andresen@meduniwien.ac.at; 7 Institute for Diagnostic and Interventional Radiology/Neuroradiology, Westkuestenklinikum Heide, Academic Teaching Hospital of the Universities of Kiel, Luebeck und Hamburg, Esmarchstraße 50, 25746 Heide, Germany; randresen@wkk-hei.de; 8 OrthoCoast, Practice for Orthopedics and Osteology, Hufelandstraße 1, 17438 Wolgast, Germany; hcr.schober@gmx.de; 9 Institute for Biostatistics and Informatics in Medicine and Ageing Research, Rostock University Medical Center, 18057 Rostock, Germany; aenne.glass@uni-rostock.de

**Keywords:** osteoporosis, finite element analysis, diffuse idiopathic skeletal hyperostosis, vertebral fracture

## Abstract

**Introduction:** Biomechanical analysis of spinal structures is crucial in the evaluation of injuries, the risk of fracture, and age-related changes. Osteoporotic vertebrae are very fragile and therefore constitute a serious risk, especially in the elderly. **Methods:** At present, clinically relevant decision making in fracture risk assessment is predicated upon finite element analysis (FEA), which utilizes high-resolution computed tomography (CT) scans from clinical practice alongside micro-CT scans from laboratory settings. Of particular interest is the utilization of cortical vertebral body thicknesses, as meticulously measured via micro-CT. The data from a body donation over 80 years old with diffuse idiopathic skeletal hyperostosis (DISH) and osteoporosis (OP) were utilized through FEA to evaluate stresses in cortical and trabecular bone and to predict the stiffness and deformability of the examined vertebral bodies. **Results:** The investigation revealed a higher density of cortical and cancellous bone in vertebrae affected by DISH. Cortical density was highest in the thoracic section (median 188 µm), while cancellous bone density was 222 HU in the cervical vertebrae. The load on cortical bone increased as we progressed towards the lumbar spine; however, it remained quite constant in cancellous bone. Despite a low bone density, we registered no fractures in vertebrae. **Conclusions:** The data showed that DISH increased the thickness of the cortical bone and the density of the cancellous bone. The combination of increased cortical and cancellous bone density might reduce the risk of fracture in patients with low bone density. These conclusions emphasize the significance of biomechanical properties in the assessment of fracture risk and have important implications for clinical practice, particularly in relation to the prevention of vertebral fractures in osteoporotic patients with DISH.

## 1. Introduction

A biomechanical evaluation of the mechanisms underlying structural alterations in the spine is of decisive importance for the proper clinical assessment of the severity of injury, the prediction of fracture risk, and for understanding the progressive aging of bone. In patients with osteoporosis (OP), it is important to evaluate the risk of fractures because they are associated with severe health-related consequences, leading to a poor quality of life, loss of independence, referral to care homes, and high morbidity and mortality rates [1]. The global incidence of clinically evident osteoporotic vertebral fractures (VFs) is about 1.4 million [2]. The tools routinely used in clinical practice are dual-energy X-ray absorptiometry (DXA) and quantitative computed tomography (QCT). However, these methods alone cannot explain all of the various strengths of vertebral bodies [3]. Finite element analysis (FEA) based on QCT images of vertebrae permits spatial visualization and could thus enhance our understanding of structural changes and their effects on the biomechanical competence of vertebrae in the presence of OP; this is just one of the many benefits of this method. To our knowledge, there are no published reports on the biomechanical behavior of vertebral bodies in all sections of the spine with the aid of FEA. Models used in the published literature have employed, to our knowledge, the values of average cortical thickness (Cr.Th) without considering the location. Although models using quantitative QCT scans have been developed to describe the geometry and density of bone in the spine [4,5,6,7], computed tomography (CT) scans lack a high degree of resolution (>0.25 mm for research purposes and >0.9 mm for clinical applications), and the cortical bone is very thin (<0.5 mm) [8,9,10]. One consequence of this is a marked overestimation of shell thickness upon clinical CT scans [9,11], which makes it difficult to incorporate the exact geometric properties of cortical bone into the model when working with clinical findings alone. Due to these difficulties, it is still not clear whether QCT-based finite element models of the spine can accurately predict biomechanical properties, such as the strength and stiffness of the entire vertebral body.

The objective of the present study was to ascertain an indication of vertebral load-bearing capacity in different segments of the spine in cases of DISH and OP. This was achieved through the integration of micro-CT-derived data on cortical thickness and trabecular structure with data from CT images of various spinal regions, utilizing FEA.

## 2. Medical History

A female body donor, aged 83, was documented with a complex medical history. She suffered from osteoporosis and diffuse idiopathic skeletal hyperostosis (DISH). Additionally, her diagnoses included type 2 diabetes mellitus, obesity, arterial hypertension, and heart failure. Her medical record also noted an unspecified stroke in her past. The deceased stood at 1.61 m (5′3″) in height and weighed 104.5 kg (230 lbs), resulting in a BMI of 40.3 kg/m^2^. This BMI categorized her as having Class III obesity. A bone density measurement of the lumbar vertebrae L1 through L3 revealed a value of 60.96 mg/cm^3^, corroborating the osteoporosis diagnosis [12]. During the examination of the body donation, it was notable that despite advanced osteoporosis, no vertebral fractures were present. Ultimately, the patient succumbed to multiple organ failure, interpreted as a consequence of generalized senility.

## 3. Methods

### 3.1. Study Design and Ethics

This clinical in vitro study was reviewed and approved by the regional ethics committee of the Medical University (ethics vote no. A 2017–0072). The body donation was part of the university’s donor program, with the patient having voluntarily consented during her lifetime for the use of her body for scientific research and education. All methods for obtaining human tissue followed the ethical guidelines outlined in the Declaration of Helsinki. The medical history was limited to the diagnoses listed in the death certificate. Vertebrae were categorized by spinal region (cervical, thoracic, and lumbar) based on their anatomical location.

### 3.2. Spines and Extraction of Vertebrae

The cadaver was perfused post mortem through the left femoral artery with a 70% ethanol solution at 0.5 bar and then preserved in a free floating state in a 0.5% aqueous phenol solution. After exposure of the migrated superficial spinal muscles (the trapezius, major and minor rhomboid muscles, levator scapulae muscle, and latissimus dorsi muscle) and the autochthonous spinal muscles, the ribs were detached paravertebrally over a hand’s breadth, exarticulated at the atlanto-occipital joint, and the ventral vertebral bodies were mobilized bluntly through the retropharyngeal space. In the region of the sacrum, approximately at the level of the sacroiliac joint space, we performed a saw cut on both sides and removed the entire spine through the dorsal aspect. The spine was then preserved in 70% ethanol at 4 °C until imaging and separation of the vertebral bodies was performed. The latter was performed from the ventral aspect by means of a cut through the intervertebral disks in the cranial and caudal aspect.

### 3.3. Diagnostic Imaging CT and QCT

**Figure 1:**
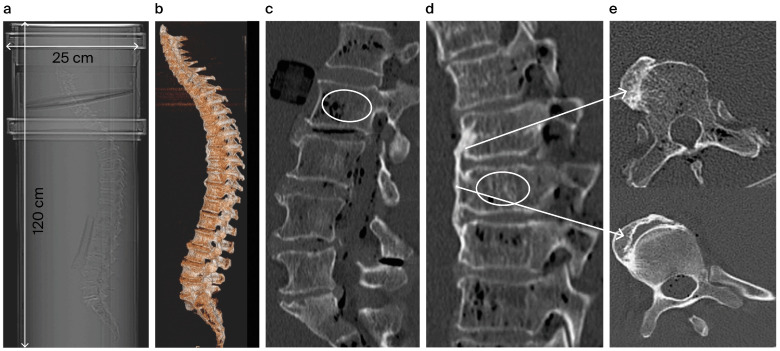
Visualization of the experimental setup on a 3D reformation of the scanned spine and positioning of the embedded spine in a PVC water phantom (**a**). For simulation of the soft tissue mantle, the spine was immersed in water and in non-aerated condition (to as great a degree as possible). The transverse diameter of the phantom is 25 cm. The entire spine in a 3D volume visualization: lateral view (**b**). Determination of the cancellous bone density in Hounsfield units (HU) using a circular region of interest (ROI) within a sagittal reformation drawn, as an example, at L2 (**c**–**e**). Figure 1d shows the anterolateral ossifications of a vertebral body affected by DISH.

To simulate a homogenous and anatomically accurate body circumference, the entire spine was placed in a plexiglass water phantom (25 cm in diameter and 125 cm in length) [13] (see Figure 1a). High-resolution whole-body CT scans (GE Revolution EVO/64-slice CT, slice thickness < 1 mm) were then acquired, with axial and sagittal reformations (slice thickness 2 mm) and 3D volume rendering [13] (see Figure 1b). Potential VFs were identified and graded by two independent radiologists using sagittal reformations [14]. A 3D volume-rendered reconstruction of the spinal anatomy was generated using an external workstation (GE AW Server ^®^ Version 2.0; GE Centricity RIS-i R version 5.0, GE Healthcare, Solingen, Germany) [13].

Cancellous bone density was measured for 22 vertebrae (from cervical vertebra 3 (C3) to lumbar vertebra 5 (L5)) in Hounsfield units (HU). The region of interest (ROI) was manually positioned in the cancellous bone, excluding cortical bone [13] (see Figure 1c–e). Mean HU values were calculated from axial, coronal, and sagittal planes, and all measurements were performed by the same radiologist to avoid intra-observer variability [13].

Bone mineral density (BMD) was further assessed using quantitative CT (GE Revolution EVO/64-slice CT and Mindways Software 3D Volumetric QCT Spine (version E8008KC), Austin, TX, USA). BMD measurements were taken from a volume block at L1, L2, and L3, and the mean value (mg/cm^3^) was used to evaluate the presence of osteoporosis [13].

### 3.4. Micro-CT Imaging

**Figure 2:**
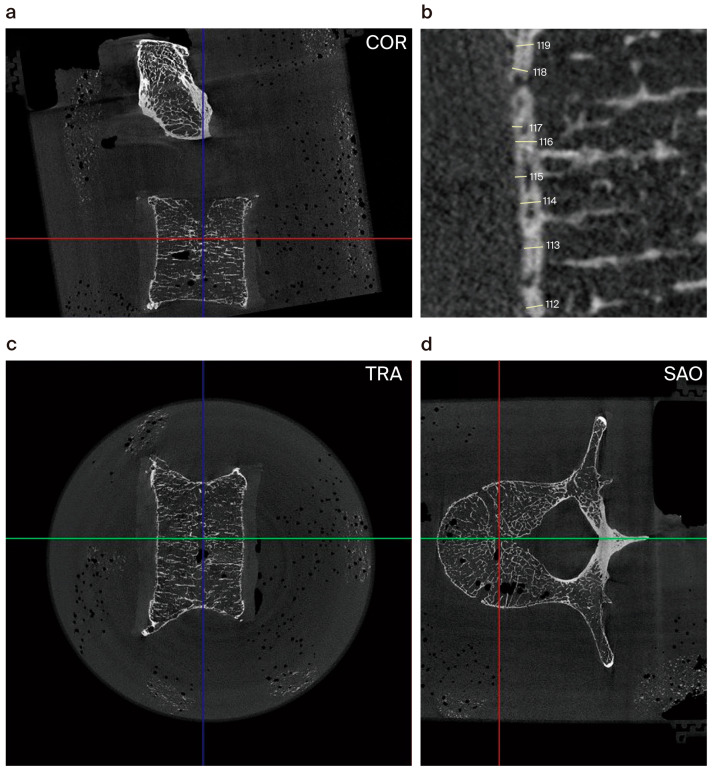
Micro-CT planes of L1; sagittal reformation (**a**) and measurement in the mid-vertebral sagittal plane (**b**). Cortical thickness is given in µm. Highest thickness is 119µm and lowest is 112µm. Examined vertebra in the frontal (**c**) and transverse planes (**d**).

Following the separation of C3 to 7, thoracic vertebrae (Th) 8 to 12, and L1 to 5 (totaling 15 of the 22 vertebral bodies), the samples were fixed in 70% ethanol within a PVC vessel and analyzed using a micro-CT device (SKYSCAN 1273, Bruker Corporation, Billerica, MA, USA) (see Figure 2a–d). Images were generated at a resolution of 4656 × 4656 pixels, with a spatial accuracy of 25 micrometers (µm). Contrast was visually established within a range of 0.001 to 0.013 absolute units (equivalent to −819 to +1340 HU).

Using the Image J software (version 1.49) (National Institutes of Health, Bethesda, MD, USA), cortical thickness (Cr.Th) was measured in micrometers at 30 points along the anterior and posterior edges (see Figure 2b). In cases of double contours caused by spongy bone within cortical bone, the outer lamella’s thickness was measured. In total, we had 1800 micro-CT data (referring to15 vertebral bodies, each measured in the ventral, dorsal, cranial and caudal region at 30 measuring points). To evaluate differences and similarities among spinal segments, we utilized balanced samples of 5 vertebral bodies per spinal section.

### 3.5. Finite Element Analysis

A biomechanical assessment of the human spine should be able to predict the deformation of and distribution of stress within individual vertebrae. It should consider the geometry of the vertebra, as reconstructed from a CT scan of the entire spine, and should include the three-dimensional form of cortical bone. As cortical thickness was not seen in adequate resolution on the CT scan, it was determined as described above on micro-CT. Cancellous bone density was determined from the CT scan data. The relevant load in this case was the weight of the upright human body. With the aid of FEA, we were able to visualize deformation and stresses in the 22 individual vertebrae. FEA is a numerical method of approximating partial differential equations in a three-dimensional area. In the present case, we solved differential equations that describe the deformation of a linear elastic body under the influence of external forces. The FEA structural analysis method has been described in detail in the existing literature [15]. The current model consists of an external surface composed of triangular shell elements. These represent cortical bone. The thickness of the shell elements is set to a constant value for a single vertebral body, but it varies between the individual vertebral bodies of the spine; in the present case, it was between 101 µm and 223 µm (Cr.Th). Tetrahedral volume elements cover the inner volume; these elements represent cancellous bone.

We chose to model the cancellous bone using volume elements with spatially averaged material properties (instead of resolving the trabecular geometry of the spongiosa) not because we lacked computing resources but because our geometrical data on the trabecular structure were incomplete. A micro-CT examination had been performed, but only on cylindrical core drills; thus, no complete 3D model of the trabecular structure was available for FE modeling.

The outer shell and the inner volume are connected by means of their common node. Every node of the lower surface of the vertebra that is in contact with the vertebra lying below it is connected through a spring element whose other end, in turn, is connected to the ground (i.e., the displacement of the grounded end is zero). This arrangement represents the elastic bed of the intervertebral disk. The elastic disks between vertebral bodies do not influence the general stress level in the bone itself. This depends merely on the external loads (in this case, the weight of the patient) and the structural properties of the vertebral body. The elasticity of the disks causes a smoothing of the local stress pattern on the lower contact surface of the body.

The determination of spring constants was based on an assumed stiffness (*Eiv*) and thickness (*tiv*) of the intervertebral disk. These properties have been described in the existing literature [16] and were assumed as follows:

(1) Eiv=9.8 MPa

(2) tiv=5 mm

All nodes on the outer surface of the vertebra that are in contact with the adjacent vertebra above it are connected to a single node located on the loading axis of the spine, through RBE3 connections, i.e., connection elements that couple a single master node to many slave nodes and distribute the force acting on the master node to the slaves without adding stiffness to the model [17]. The resulting force is then applied to this individual node in the direction of the loading axis. 

The load axis was placed normal to and through the center of the upper contact surface of the vertebral body. The magnitude of the load force was dependent on the location within the spine of the vertebral body being considered and the overall body mass of the patient. The actual force was computed according to the distribution outlined by Duval-Beaupere et al. [18].

First, the shape of the vertebral body was reconstructed as a 3D surface from the existing CT scan data with the aid of the 3D Slicer software package (version 5.6.2) [19]. The process yielded triangulated surfaces on the outer shell of the vertebral body. As the shape of the triangles in a mesh of this type are unsuitable for direct integration into an FEA mesh, the following procedure was used to produce the mesh.

1. The vertebra was meshed using the open source software package Snappy Hex-Mesh (version 12) (OpenFOAM Open CFD Ltd., Bracknell, UK, 2024) [20]. This software package has consistently high-quality input geometry and is able to re-mesh triangulated surfaces.

2. The resulting polyhedral mesh was then triangulated and converted into an FEA mesh. The FEA solution was generated with the aid of the finite element software (Code Aster, version 13.3) [21]. In addition to the geometric mesh, the FEA model also requires material properties. The properties of cortical bone were set to a constant, referred to as Ecor [22]:

(3) Ecor=8000 MPa

The properties of cancellous bone were set to a spatially uniform value. This value was derived from CT data by first determining the mean Hounsfield value of the cancellous bone volume. From this Hounsfield value, the elasticity module (*E*) and the maximal tolerated stress of cancellous bone (*Smax,spong.*) were determined from the HU value for all points inside the vertebral body. To determine other properties, we first calculated bone mineral density (*ρBMD*), as follows:

(4) $$\rho_{BMD}=a+max*b$$

The following values were established for the operators: *a* = −14 mg/cm^3^, *b* = 0.385 mg/cm^3^/HU, and a minimal Hounsfield value (HUmin = 50). 

Then, bone ash density was calculated as follows:

(5) $$\rho_{CHA}=max\left(0,\;D_{CHA}\frac{\rho_{BMD}-D_{H_{2}O}}{D_{CHA}-D_{H_{2}O}}\right)$$

(6) $$\rho_{ash}=0.0633\frac{g}{{cm}^{3}}+0.887\;\rho_{CHA}$$

with *D_H_*_2*O*_ = 1 g/cm^3^ and *D_CHA_* = 3.18 g/cm^3^. 

Finally, we generated the *E* module and the maximal stress for each point [23]:

(7) $$E_{spong}=21,700\;MPa{\left(\frac{\rho_{ash}}{g/{cm}^{3}}\right)}^{2.07}$$

(8) $$S_{max,\;spong}=137\;MPa\;{\left(\frac{\rho_{ash}}{g/{cm}^{3}}\right)}^{1.88}$$

### 3.6. Statistics

This case report is based on statistics derived from several measurements of 22 individual vertebral bodies (C3–7, Th1–12, L1–5). Descriptive statistics of quantitative variables, such as cancellous bone density, cortical thickness, and stress placed on bone, are presented in median values with the respecting quartiles (Q1–Q3). The data were analyzed using non-parametric test methods such as the Kruskal–Wallis test for differences between three spinal sections (cervical spine CS, thoracic spine TS, and lumbar spine LS), or the four investigated regions of vertebral bodies (ventral, dorsal, cranial, and caudal), respectively, and subgroups underwent pairwise post-testing, with the U-test. Bonferroni-adjusted *p*-values are given.

For the purpose of illustrating subgroup differences in measured cortical thickness (µm), FEA-simulated stress levels (in MPa), and deflection (mm), we estimated the 95% confidence intervals (95% CI) of mean values. 

The correlation between cancellous bone density (HU) and cortical thickness (µm) was analyzed, and we tested the Spearman correlation coefficient, r_S,_ to be zero. 

All data were analyzed using the statistical software package IBM^®^ SPSS^®^ 29.0. We considered *p*-values of *p* < 0.05 resulting from two-sided tests to be statistically significant.

## 4. Results

We investigated an entire spine, evaluating the CT-derived data of 22 vertebrae (C3–7, Th1–12, L1–5) and the micro-CT-derived data of 15 vertebrae (C3–7, Th8–12, L1–5) extracted from a body donor (83 years old, female).

### 4.1. CT/QCT

The CT examination revealed intriguing findings. In the thoracic spine region, specifically between Th5 and Th8, a pronounced endosclerosis was observed. This was accompanied by an increased density of the cancellous bone. Notably, there was continuous ossification along the anterolateral aspects of these vertebrae. Curiously, the intervertebral disc heights were preserved in this case. Close inspection showed no evidence of ankylosis in the facet joints, nor any involvement of the sacroiliac joints. This constellation of findings strongly suggested a characteristic presentation of DISH [24]. The juxtaposition of bony changes with maintained disc spaces and the absence of joint involvement are hallmarks of DISH [24], distinguishing it from other degenerative or inflammatory spinal conditions. This case exemplifies the importance of meticulous radiological analysis in differentiating various spinal pathologies. The observed endosclerosis and increased trabecular density, coupled with the flowing ossification pattern, paint a vivid picture of the skeletal changes associated with DISH. These findings not only confirm the diagnosis but also provide valuable insights into the progression and manifestation of this condition in the thoracic spine.

**Figure 3:**
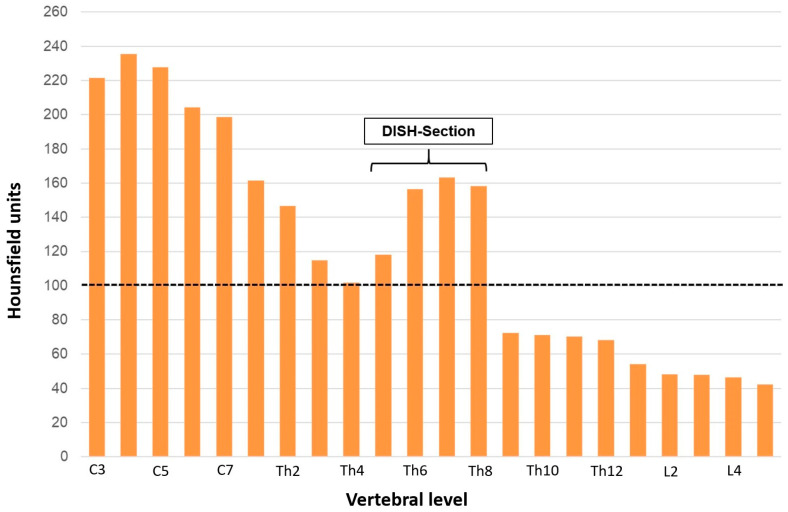
Cancellous bone density in Hounsfield units (HU) determined for the 22 individual vertebral bodies (C3-L5). The estimation of osteoporosis using Hounsfield units depends on the age of the patient and the site of the measurement. Generally, patients aged around 60 years with a value of around 100 HU in the lumbar spine have an age-appropriate cancellous bone density. In patients in their 80 s, values below 65 HU are consistent with osteoporosis. The area between vertebral bodies Th5 and Th8 is marked by endosclerosis due to diffuse idiopathic skeletal hyperostosis (DISH).

The examination of a deceased body donor yielded noteworthy insights into her bone health and physical constitution. The LS exhibited an average bone mineral density (BMD) of approximately 61 mg/cm^3^, indicative of established OP. Intriguingly, despite these low BMD values, no signs of vertebral fractures were detected in the axial skeleton. The donor presented with a substantial body mass index (BMI) of about 40 kg/m^2^, suggesting significant obesity. In addition to the BMD measurement, the density of cancellous bone was quantified using HU. The density of cancellous bone in the cervical vertebrae (222 HU; 202–232) was significantly higher than that in the thoracic (117 HU; 71.4–158; *p* < 0.001) or lumbar vertebrae (47.8 HU; 44.3–51.2; *p* = 0.024) of the investigated spine. The density of thoracic and lumbar cancellous bone also differed (*p* < 0.001). The distribution of mid-vertebral cancellous bone density throughout the entire spine revealed high values in the CS and in the thoracic section affected by DISH (see Figure 3). The HU values determined in the present case were not correlated with cancellous bone density in the respective vertebra (r_S_ = 0.236, *p* > 0.05).

### 4.2. Micro-CT

The cortical thickness of spinal sections determined by micro-CT differed significantly (*p* = 0.003) between the thoracic (188 µm; 164–221) and lumbar spine (149 µm; 129–171). In contrast, the thickness of the cervical spine (156 µm; 126–212) was not significantly different from both the thoracic and lumbar spine.

Thickness in the cortical regions and endplates was lowest in the dorsal region (152 µm; 134–174). It differed most prominently from the ventral region (186 µm; 130–230; *p* = 0.048), and to a lesser degree from the caudal (185 µm; 144–214) and cranial regions (168 µm; 134–196).

**Figure 4:**
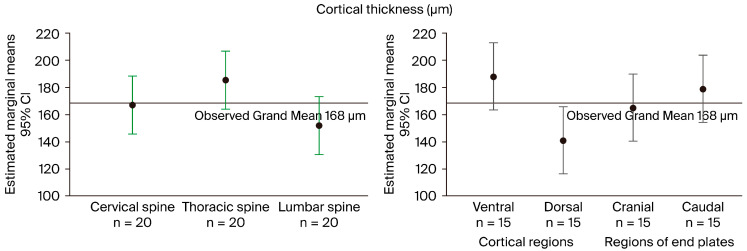
Illustration of subgroup differences in the estimated cortical thickness from the observed mean level (168 µm, black reference line) in this case (advanced age, OP, DISH). **Left** (green): above-average cortical thickness was noted in the thoracic spine section (mean: 186 µm) and below-average thickness in the lumbar section (mean: 152 µm). Cortical thickness was measured in 15 vertebral bodies in total, i.e., 5 per spinal section (cervical C3–7, thoracic Th8–12, lumbar L1–5), with each vertebral body measured in 4 regions: 2 cortical and 2 endplates (n = 20). **Right** (gray): the most pronounced difference we observed was between the measured cortical thickness values of the ventral (mean: 188 µm) and dorsal (mean: 140 µm) bodies (n = 15 vertebral bodies).

Overall, the observed grand mean cortical thickness of 168 µm in the reported case (83 years, OP, DISH) was low compared to the nearly two-fold higher cortical thickness of about 300 µm observed elsewhere. Figure 4 illustrates various features of this case: cortical thickness in the three spinal sections (left panel) and four investigated regions of the vertebral body (right).

### 4.3. Finite Element Analysis

**Figure 5:**
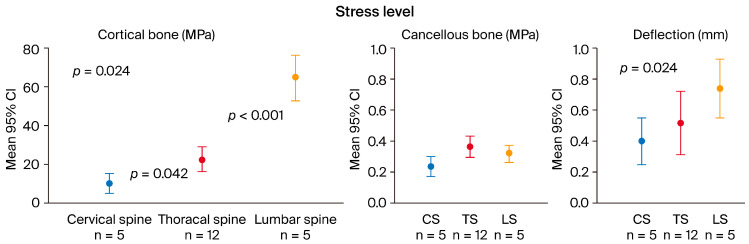
FEA-simulated stress levels (MPa) on cortical (**left panel**) and cancellous bone (**middle panel**) with reference to spine sections: cervical section CS (n = 5 vertebral bodies), thoracic section TS (n = 12), and lumbar section LS (n = 5). We noted a greater than 6-fold significant increase in stresses on cortical bone in the craniocaudal direction, while stresses on cancellous bone remained at a similar level. The **right panel** shows the deflection (mm) of the vertebrae; the maximum deflection in the lumbar spine differs significantly from the deflection in the cervical section.

Stresses in the cortical bone differed in the three sections of the spine. It was lowest in the cervical region (10.4 MPa; 6.05–13.5) and increased in the thoracic (22.4 MPa; 14.2–36.1) and lumbar regions (67.2 MPa; 54.7–73.0). The differences in cortical stress between the spinal sections were found to be statistically significant: CS vs. TS *p* = 0.042; CS vs. LS *p* = 0.024; TS vs. LS *p* < 0.001 (see Figure 5, left panel).

Stresses on cancellous bone, on the other hand, were similar in all three sections of the spine (0.20 MPa, 0.35 MPa, 0.30 MPa) and markedly lower (see Figure 5, middle panel).

**Figure 6:**
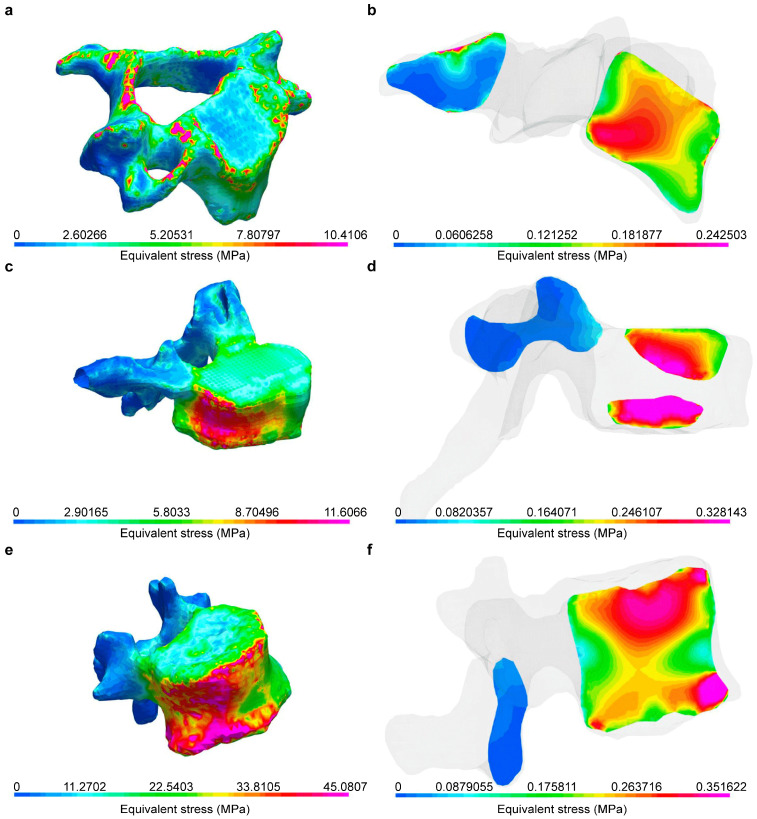
Finite element modeling of stresses on cortical and cancellous bone in vertebral bodies C3 (**a**,**b**), Th8 (**c**,**d**), and L1 (**e**,**f**). Stresses on cortical and cancellous bone increased from the cranial to the caudal aspect, as color-coded herein from blue (0 MPa) to pink (peak stress).

The maximal deflection of the vertebrae was greatest in the lumbar region (0.70 mm; 0.65–0.85 mm) and differed significantly from the deflection of the cervical (0.40 mm; 0.30–0.50 mm; *p* = 0.024) but not the thoracic (0.45 mm; 0.30–0.58 mm) vertebrae (see Figure 5, right panel). In Figure 6, finite element modeling of individual vertebral bodies from the cervical, thoracic, and lumbar spine (C3, Th8, L1) are presented as illustrative examples. The highest stresses (highlighted in pink) are observed in the regions of the cortical ring and the endplates. This visualization demonstrates the distribution of mechanical stress across different spinal segments. The concentration of peak stresses in the cortical shell and endplates is consistent with their crucial role in load-bearing and force transmission within the vertebral structure.

## 5. Discussion

### 5.1. Discussion of the Method

We have utilized this case to present an initial calculation of the biomechanical competence of vertebral bodies from various spinal segments using FEA. For this purpose, we incorporated individually measured cortical thicknesses from micro-CT with trabecular densities from CT data. To obtain maximally diverse data, we selected an older female body donor with DISH and osteoporosis. This allowed for a comprehensive overview of the biomechanical properties of the examined vertebral bodies, particularly regarding failure load, failure stress, and degree of deflection. To estimate the trabecular density of the vertebral bodies, we determined HU values via standard CT using a circular ROI, alongside QCT measurements. Ours is therefore an objective and standardized method of measuring bone density that can be directly integrated into the FE model in a clinical setting. This method is highly applicable [25], has low interobserver variability [26], and can be outputted by CT scanners operating in peripheral hospitals.

Determination of cortical thickness using micro-CT offers very high resolution and accuracy in capturing the structure of bone. This method, in turn, improves our predictions of failure load, failure stress, and the degree of deflection via FE analysis. This method was chosen because the resolution of the cortex upon standard CT examination is often insufficient. Moreover, cortical density decreases less with age than trabecular density does, which we accounted for the present study. Some FE studies stipulate a standardized value of cortical ring thickness; this can place excessively high or low failure loads and stresses upon spinal segments. In our study, patient-specific modeling was utilized. Furthermore, the decrease in bone density in osteoporosis and its increase in DISH were to be represented in the FEA. By using CT and micro-CT data, the aim was to create a highly individualized model that considers the specific anatomical features of the examined body donor. Accurately determining bone quality is crucial for successful treatment that will be prevent osteoporotic fractures and optimally prepare patients for surgical spinal procedures [27].

### 5.2. Discussion of Disease-Specific Aspects of the Present Case

In this study, we chose to examine the spine of an 83-year-old female body donor diagnosed with diffuse idiopathic skeletal hyperostosis (DISH) and osteoporosis (OP). The donor had a notably high BMI of 40.3 kg/m^2^, which is linked to a variety of comorbidities, including type II diabetes, arterial hypertension, hypoventilation syndromes, and non-alcoholic fatty liver disease [28]. The presence of metabolic and cardiovascular comorbidities is frequently observed in patients with DISH [29,30,31,32], as was the case here. These conditions can influence bone metabolism and density. While excess weight is sometimes associated with a reduced fracture risk [33,34], other studies suggest an increased risk of fractures in obese individuals [35]. The body donor had a bone mineral density (BMD) below 80 mg/cm^3^, indicating osteoporosis [36]. Typically, BMD values below 60 mg/cm^3^ are linked to fusion fractures [37], yet no fractures were found in the spine of this donor, despite her advanced age, which is a significant risk factor. Fracture rates increase substantially after the age of 70 [38], particularly in Th7, 8, and 12, as well as L1 and 2 [25,39]. Interestingly, the donor showed increased cancellous bone density between Th5 and 8 (vertebral bodies affected by DISH), which may have helped prevent pathological fractures.

The literature on BMD in patients with DISH compared to healthy controls is inconsistent, varying depending on the measurement methods used. While Diederichs et al. [40] found higher BMD in men with DISH compared to those without, this was not reflected in quantitative computed tomography (QCT) results. However, both DXA-BMD and QCT-BMD values were notably higher in individuals with severe lumbar DISH [40]. Our donor exhibited significantly higher cortical bone density in the thoracic region. Some authors have described a biphasic pattern in both healthy individuals and those with OP, where higher values are observed in the cervical spine, a drop in the transition to the thoracic spine, followed by an increase in the lumbar spine [41,42]. This pattern was also observed in our case, with the lowest cortical density found in the LS. Unlike Ritzel et al. [41,42], who focused on the ventral and dorsal cortical bone, we found a similar and lower curve for dorsal cortical bone across spinal sections. This difference could be attributed to the age of the body donor, who was, on average, 6 years older than the subjects in the previous studies. Additionally, those studies did not include the endplates in their evaluation, whereas we did.

The higher density of cortical bone in the ventral region, compared to the dorsal, may protect against endplate fractures. For example, Silva et al. [9] reported a mean cortical thickness of about 350 µm at L1 in individuals over the age of 40, significantly higher than what we found in our donor (83 years old). These findings suggest that cortical bone thickness may be age-dependent [10]. Age-related thinning of cortical bone has been documented in women with OP [43]. Silva et al. [9] also observed that in the mid-sagittal plane of L1, the ventral cortical ring is thicker than the dorsal ring and the endplates. In our study, the posterior edge was consistently thinner than the endplates.

Andresen et al. [44] concluded that both cancellous and cortical bone contribute to vertebral load-bearing capacity. In healthy aging, the weakening of cancellous bone leads to increased reliance on cortical bone for structural support. In our case, the thoracic region exhibited the strongest cortical and cancellous bone, likely due to DISH. FEA also confirmed the higher load-bearing capacity of cancellous and cortical bone in the thoracic vertebrae. Osteoporotic insufficiency fractures do not occur in the CS. At this site, the stress on cortical and cancellous bone is minimal. Concurrently, the maximal deflection of vertebrae in the CS is also less pronounced. Whether increased bone density or improved bone quality is responsible for the present results cannot be conclusively determined. Bone density and bone quality are two distinct yet interrelated concepts, both being crucial for overall bone strength and health. Bone density refers to the ratio of mineralized bone to the defined bone volume, while bone quality is defined by the bone’s microarchitecture. The structural changes that we observed in cortical and cancellous bone explain how the spine is protected from VFs and were further proven by the FEA.

## 6. Limitations

The primary weakness of this study lies in the limited sample size of only one cadaveric specimen, which significantly restricts the generalizability of the results. Without comparative data from other cadaveric specimens or in vivo measurements, validating the model is hardly feasible. Furthermore, age- and sex-specific variations cannot be accounted for, thereby limiting the transferability of our data to other demographic groups. The static nature of FE modeling potentially neglects important dynamic aspects of spinal loading in daily life. Despite high-resolution imaging, certain fine structures or material properties may have been simplified or neglected in the model, which may have affected the accuracy of our results. Finally, our focus on osseous structures may have caused the influence of soft tissues (such as intervertebral disks, ligaments, and muscles) on failure loads and stress to be underestimated. In future investigations, a larger and more diverse donor group should be studied, so that we might better represent inter-individual variability and enhance the generalizability of the results. Moreover, the determination of cortical thickness using micro-CT examinations is experimental in nature and is mostly performed through in vitro measurements on deceased individuals; this should also be considered a limitation. The integration of in vivo measurements and dynamic loading scenarios could improve the models’ fidelity to reality.

## 7. Conclusions 

The findings of the present study suggest that DISH may exert a protective effect against vertebral fractures even in the presence of osteoporosis. The following points could be of significance for clinical practice:

DISH exhibits a dual effect on vertebral bone structure; it not only enhances the thickness of the cortical bone, as anticipated, but also elevates the density of the cancellous bone within the affected vertebrae. 

The highest thickness of cortical bone in the donor’s body was observed within the vertebrae of the TS.

The thickness of the cortical bone varies across different segments of the spine.

The cortical thickness is lowest in the posterior region of the vertebral body.

Regardless of the section of the spine, the highest cortical density is found in the ventral aspect of the vertebral body. 

The highest density of cancellous bone was found in the cervical vertebrae.

As one proceeds in the craniocaudal direction, stress placed on cortical bone increases; such stress remains at a similar level in cancellous bone throughout the sections of the spine.

It is possible that in the current case report, DISH had a protective effect on the occurrence of vertebral fractures in the presence of osteoporosis.

## Data Availability

The data presented in this study are available upon request from the corresponding author.

## Abbreviations

BMD	bone mineral density (mg/cm^3^)
cm	centimeter
Corp.	corporation
Cr.Th	cortical thickness (µm)
CI	confidence interval
CS	cervical spine
CT	computed tomography
C1–7	cervical vertebra 1–7
DISH	diffuse idiopathic skeletal hyperostosis
DXA	dual-energy X-ray absorptiometry
Ecor	stiffness of the cortical bone
Eiv	stiffness of the intervertebral disk
FEA	finite element analysis
g/cm^3^	gram/cubic centimeter
GE	General Electric
HU	Hounsfield units
IBM	International Business Machines Corporation
i.e.,	that’s is
kg	kilogram
kg/m^2^	kilogram/square meter
Ltd.	Limited
LS	lumbar spine
L1–5	lumbar vertebra 1–5
m	meter
mm	millimeter
mg/cm^3^	milligram/cubic centimeter
MPa	mega pascal
n	number (count)
no.	number
OP	osteoporosis
PVC	polyvinyl chloride
Q1–Q3	quartile 1 to 3
QCT	quantitative computed tomography
ROI	region of interest
tiv	thickness of the intervertebral disk
Th1–12	thoracic vertebra 1–12
TS	thoracic spine
USA	United States of America
vcor	thickness of cortical bone
VFs	vertebral fractures
µm	micrometer
°C	degrees Celsius
